# Abscisic Acid Stimulates Ethylene Biosynthesis by Repressing the Expression of *FaMADS1* in Postharvest Strawberry Fruit

**DOI:** 10.3390/plants15142202

**Published:** 2026-07-19

**Authors:** Renchi Chen, Yuhua Yan

**Affiliations:** 1 Institute of Food Science, Wenzhou Academy of Agricultural Science, Wenzhou 325006, China; yanyuhua@wzvcst.edu.cn; 2 Zhejiang Key Laboratory of Agri-Food Resources and High-Value Utilization, Wenzhou 325006, China; 3 Southern Zhejiang Key Laboratory of Crop Breeding, Wenzhou Academy of Agricultural Science, Wenzhou 325006, China

**Keywords:** abscisic acid, *FaMADS1*, ethylene biosynthesis, strawberry fruit ripening

## Abstract

Abscisic acid (ABA) and ethylene are both essential regulators of strawberry fruit (*Fragaria* × *ananassa* Duch.) ripening. This study investigated the interplay between ABA and ethylene, revealing that ABA downregulates *FaMADS1*, thereby de-repressing ethylene biosynthesis genes. Functional validation via *FaMADS1* silencing and over-expression confirmed that *FaMADS1* acts as a negative regulator of ethylene biosynthesis key genes (*FaSAMS1*, *FaACS1*, and *FaACO1*). Furthermore, dual-luciferase assays demonstrated *FaMADS1*’s direct suppression of the promoters of key ethylene biosynthesis genes. Collectively, these results elucidated a novel mechanism wherein ABA and ethylene coordinate ripening via *FaMADS1*-mediated transcriptional repression. This ABA-*FaMADS1*-ethylene regulatory cascade provides a theoretical basis for targeted postharvest regulation of strawberry ripening, laying a foundation for developing low-cost, hormone-based preservation technologies and molecular breeding strategies to alleviate rapid quality deterioration and economic losses of harvested strawberry.

## 1. Introduction

The maturation of fleshy fruits represents a complex regulatory process characterized by coordinated modifications in pigmentation patterns and tissue-softening dynamics [1,2,3,4]. Recent molecular investigations have substantially advanced our comprehension of non-climacteric ripening physiology, particularly through model species studies [5,6,7]. Among these, the octoploid strawberry (*Fragaria* × *ananassa* Duch.) serves as an exemplary model organism, valued globally due not only to its vibrant pigmentation and palatable flavor profile but also its rich phytochemical profile [8,9,10]. For strawberry, ripening is far more than the visible changes of red coloration and fruit softening that are commonly used as simple ripening markers. A complete ripening process also encompasses profound changes in soluble sugar composition, organic acid content, sugar–acid ratio and volatile flavor compounds, which are the core determinants of fruit taste, flavor and overall sensory quality [11]. These quality traits directly govern consumer preference and repeat purchase willingness and are critical commercial indicators for fresh berry production. As a typical highly perishable non-climacteric fruit, strawberry suffers from rapid quality deterioration, water loss and rot after harvest, resulting in short shelf life and severe economic losses [12]. Therefore, exploring regulatory mechanisms of fruit ripening and developing strategies to maintain comprehensive quality during postharvest storage have long been research hotspots in postharvest biology [13].

The strawberry fruit-ripening process is synergistically regulated by multiple phytohormones, including abscisic acid (ABA), indoleacetic acid (IAA), and ethylene [14,15,16,17]. Specific biological functions operating through a network of different phytohormones are related in controlling strawberry fruit ripening. A previous study suggested that IAA was a vital part of inhibiting strawberry fruit ripening and can delay the accumulation of anthocyanin, decrease in firmness, and loss of chlorophyll [18,19]. Contrarily, ABA was considered to have a positive effect on strawberry fruit ripening. Symons et al. observed that the ABA content continually increased during the process of strawberry fruit ripening [20]. ABA accelerated anthocyanin accumulation and fruit firmness decline to promote strawberry ripening [11,21]. Accumulating evidence has proven that exogenous ABA modulates multiple metabolic pathways associated with fruit quality and storage performance in postharvest strawberry. A recent study demonstrated that low-concentration ABA treatment could prolong postharvest life of strawberry via regulating sucrose metabolism and maintaining cell wall integrity; ABA significantly increased the contents of soluble sucrose and glucose and suppressed the expression of cell wall degradation-related enzymes to retard fruit deterioration during cold storage [11]. Additionally, ABA is tightly linked to the synthesis of flavor volatiles and the balance of sugar and acid in ripening fruits, jointly shaping the edible quality of strawberry [22,23]. Emerging evidence highlights ABA as a central coordinator of metabolic reprogramming, particularly in enhancing soluble carbohydrate biosynthesis, chromoplast differentiation, and volatile compound biosynthesis [5,24,25]. Contemporary studies have revealed ethylene’s previously underestimated regulatory capacity in non-climacteric fruit maturation. Traditionally, ripening of non-climacteric fruits was considered largely independent of ethylene and mainly governed by ABA signals, owing to the absence of a typical respiratory and ethylene burst during ripening progression. However, a recent comprehensive overview systematically summarized accumulating genetic and physiological evidence proving ethylene acts as an indispensable ripening regulator across various non-climacteric crops, including strawberry, by modulating pigment accumulation, cell wall degradation and postharvest storability [26]. Experimental evidence demonstrated that ethylene signaling modulates multiple ripening-related pathways, including anthocyanin biosynthesis activation, cell wall hydrolase induction, and chlorophyll degradation mechanisms [27,28,29]. These findings collectively establish ABA and ethylene as synergistic regulators orchestrating the ripening network in strawberry fruit.

To date, there is accumulating evidence indicating that ethylene biosynthesis might be induced by ABA in strawberry fruit. For instance, different concentrations of ABA solution could enhance the ethylene production of ‘Everest’ strawberry fruit [30]. ABA treatment promoted ethylene production in ‘Akihime’ strawberry fruit [31]. The transcriptomic analysis of ‘Toyonoka’ strawberry showed that ABA obviously up-regulated ethylene biosynthesis-related gene *FaACO3* expression, while control fruit displayed no significant difference [32]. Three pivotal genes, *FaSAMS1*, *FaACS1* and *FaACO1,* govern successive steps of ethylene biosynthesis: *FaSAMS1* synthesizes ethylene precursor SAM, *FaACS1* catalyzes SAM into ACC, and *FaACO1* converts ACC to ethylene. Elevated transcription of these three genes collectively accelerates ethylene generation in strawberry fruit [33,34]. These studies suggest that strawberry fruit-ripening processes are highly coordinated between ABA and ethylene.

Transcriptional regulation is fundamental to ABA-inducible gene expression governing strawberry fruit ripening. Within this regulatory network, MADS-box transcription factors (TFs) serve as critical modulators of the ABA signaling pathway [35]. Supporting this, ABA treatment down-regulated the C-type MADS-box gene *FaSHP* in ‘Elsanta’ strawberry fruit [36], while the expression patterns of *FaMADS4* and *FaMADS9* correlate with endogenous ABA levels [37]. Building on these findings, our prior research identified *FaMADS1* as an ABA-repressed transcriptional suppressor that modulates ripening [19,38].

Although the roles of ABA and ethylene in regulating strawberry sugar metabolism, cell wall metabolism and shelf life have been well-documented, the upstream molecular mechanism by which ABA triggers ethylene biosynthesis remains unclear. Moreover, how the ABA-*FaMADS1*-ethylene regulatory cascade interacts with sugar-acid metabolism, flavor formation and postharvest storage performance, and further affects consumer acceptance of strawberry fruit, still requires systematic exploration. Based on the above research gaps, we proposed two central hypotheses for this work: first, ABA signaling upregulates ethylene biosynthesis in postharvest strawberry by repressing the transcription factor *FaMADS1*; second, *FaMADS1* directly binds and inhibits the promoters of three key ethylene biosynthetic genes (*FaSAMS1*, *FaACS1*, *FaACO1*) to restrict ethylene accumulation. This study systematically verifies the above hypotheses via physiological treatments, gene transient transformation and dual-luciferase reporter assays. With these mechanistic insights, we propose a novel postharvest biotechnology approach: precision modulation of the ABA-*FaMADS1* node could decouple ripening-associated metabolic processes, thereby establishing molecular basis for prolonging fruit commercial viability through targeted transcriptional engineering.

## 2. Results

### 2.1. Phenotype, Ethylene Production, Fruit Color, Firmness, and Total Anthocyanin Content (TAC) Change in Strawberry Fruit

**Figure 1:**
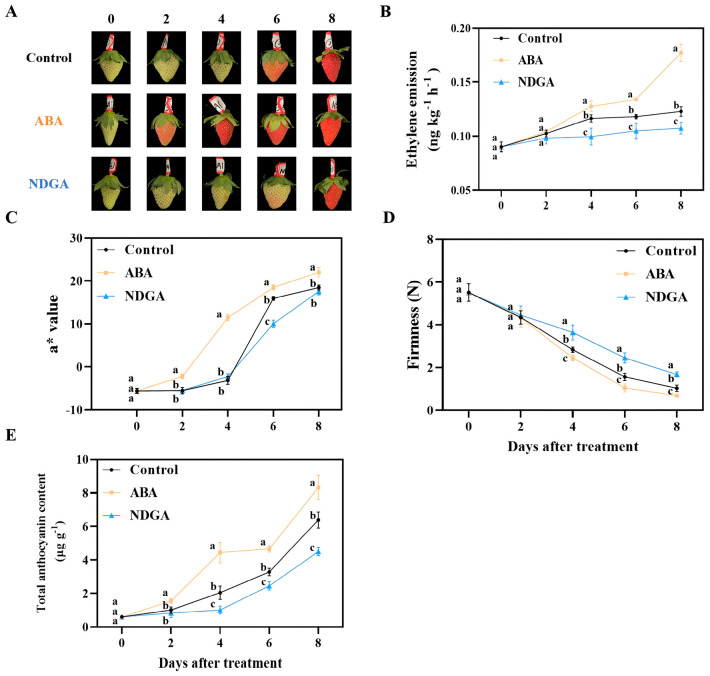
Phenotype (**A**), ethylene emission (**B**), a* value (**C**), firmness (**D**), and total anthocyanin content (**E**) change of strawberry fruit. Error bars represent standard deviation (SD) of triplicate biological replicates (n = 3). Prior to Duncan’s multiple range test, Shapiro–Wilk normality test and Levene’s homogeneity of variance test were conducted; all data met normality and homoscedasticity requirements. Significant differences (*p* < 0.05) between treatment groups were determined using Duncan’s multiple range test, denoted by distinct lowercase letters (a–c).

Strawberry fruit ripening, assessed by color shift (white to red), was accelerated by ABA treatment and inhibited by NDGA over days 2–8, with significant color differences apparent at day 6 after treatment (Figure 1A). Ethylene production revealed progressive elevation from days 2 to 4 and a sharp upsurge from days 6 to 8 in ABA-treated strawberry fruit. Ethylene output diverged substantially between ABA and NDGA treatments during days 6–8 (Figure 1B). For the chromatic parameter a*, ABA induced a rapid rise (days 2–4) followed by a steadier increase (days 4–8). A significant disparity in a* value emerged between the ABA and NDGA groups after day 6 (Figure 1C). Fruit firmness decreased under ABA but was maintained at higher levels with NDGA (Figure 1D). Total anthocyanin content (TAC) responded positively to ABA and negatively to NDGA. Post-6-day treatments yielded TAC values of 150% (ABA) and 75% (NDGA) relative to controls (Figure 1E).

### 2.2. Expression of FaMADS1 and Ethylene Biosynthesis-Related Genes

**Figure 2:**
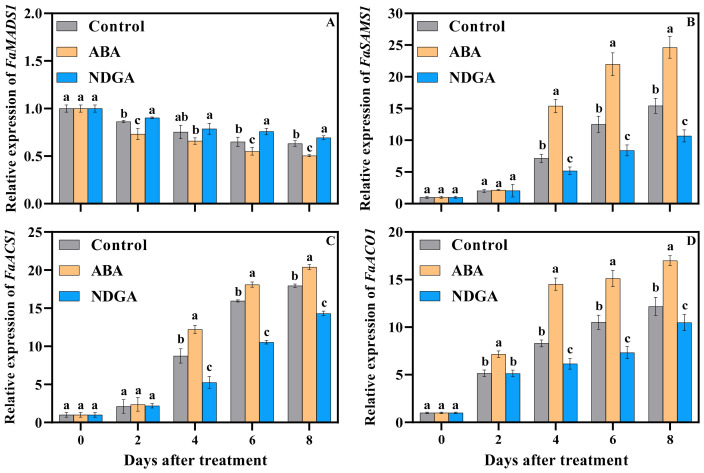
Relative expression of *FaMADS1* and ethylene biosynthesis-related genes in strawberry fruit. (**A**) *FaMADS1*, (**B**) *FaSAMS1*, (**C**) *FaACS1*, and (**D**) *FaACO1*. Error bars represent standard deviation (SD) of triplicate biological replicates (n = 3). Prior to Duncan’s multiple range test, Shapiro–Wilk normality test and Levene’s homogeneity of variance test were conducted; all data met normality and homoscedasticity requirements. Significant differences (*p* < 0.05) between treatment groups were determined using Duncan’s multiple range test, denoted by distinct lowercase letters (a–c).

To elucidate *FaMADS1*’s interaction with ethylene biosynthesis, we analyzed transient expression of *FaMADS1*, *FaSAMS1*, *FaACS1*, and *FaACO1* (Figure 2). *FaMADS1* decreased progressively over 8 days, with ABA treatment yielding significantly lower expression than NDGA (0.7-fold vs. 1.2-fold control at day 8; Figure 2A). By day 8, *FaMADS1* expression in the ABA and NDGA treatment groups was 0.7-fold and 1.2-fold of the control levels, respectively (Figure 2A). Conversely, *FaSAMS1* expression increased post-treatment in all groups. ABA elevated *FaSAMS1* to 1.91-fold control levels at day 8, while NDGA resulted in only 0.69-fold expression (Figure 2B). ABA strongly up-regulated *FaACS1* relative to the control, whereas NDGA suppressed it. *FaACS1* expression surged sharply from days 6 to 8 under ABA but increased gradually with NDGA (Figure 2C). *FaACO1* exhibited a parallel expression pattern to *FaACS1* (Figure 2D).

### 2.3. FaMADS1 Inhibition of Ethylene Biosynthesis

**Figure 3:**
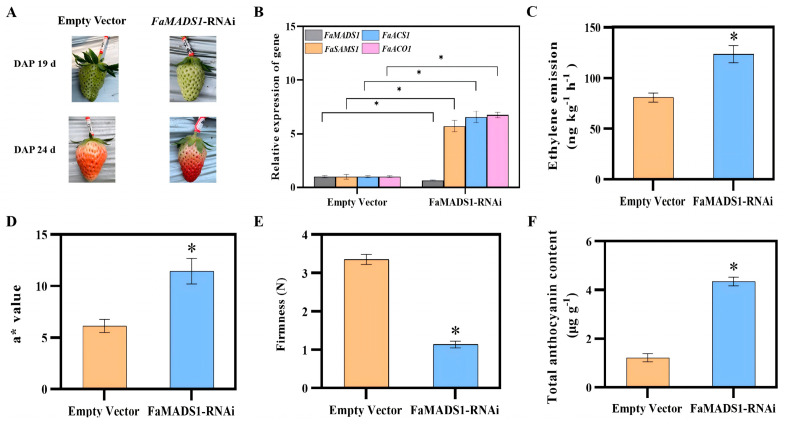
Influence of *FaMADS1* silencing on ethylene biosynthesis, color, and firmness of strawberry fruit. (**A**) Fruit appearance; (**B**) relative expression of genes; (**C**) ethylene emission; (**D**) a* value; (**E**) fruit firmness; (**F**) total anthocyanin content. Error bars represent standard deviation (SD) of triplicate biological replicates (n = 3). Prior to Duncan’s multiple range test, Shapiro–Wilk normality test and Levene’s homogeneity of variance test were conducted; all data met normality and homoscedasticity requirements. Significant differences (*p* < 0.05) between treatment groups were determined using Duncan’s multiple range test, denoted by asterisk (*).

**Figure 4:**
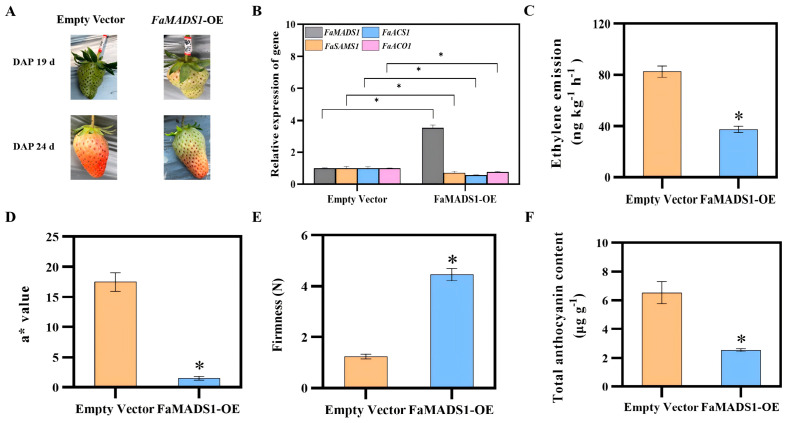
Influence of *FaMADS1* over-expression on ethylene biosynthesis, color, and firmness of strawberry fruit. (**A**) Fruit appearance; (**B**) Relative expression of genes; (**C**) Ethylene emission; (**D**) a* value; (**E**) Fruit firmness; (**F**) Total anthocyanin content. Error bars represent standard deviation (SD) of triplicate biological replicates (n = 3). Prior to Duncan’s multiple range test, Shapiro–Wilk normality test and Levene’s homogeneity of variance test were conducted; all data met normality and homoscedasticity requirements. Significant differences (*p* < 0.05) between treatment groups were determined using Duncan’s multiple range test, denoted by asterisk (*).

Functional evidence for *FaMADS1*-mediated repression of ethylene production was obtained through transient silencing and over-expression in white-stage (19 DAA) strawberry fruit. Silencing accelerated fruit reddening (Figure 3A), while over-expression inhibited color development (Figure 4A). Molecular analyses confirmed *FaMADS1* knockdown (0.33-fold vs. control; Figure 3C) and over-expression (2.52-fold vs. empty vector control; Figure 4C). Elevated ethylene emission in silenced fruit (Figure 3B) correlated with induced expression of *FaSAMS1* (1.35×), *FaACS1* (2.16×), and *FaACO1* (1.35×) versus the control (Figure 3B). Reciprocally, suppressed ethylene synthesis in over-expressing fruit (Figure 4B) aligned with reduced transcript levels of *FaSAMS1* (0.71×), *FaACS1* (0.57×), and *FaACO1* (0.75×) relative to empty vector controls (Figure 4B).

Additionally, *FaMADS1* silencing induced higher a* value and total anthocyanin accumulation (Figure 3D,F), and the control fruit maintained particularly higher firmness (Figure 3E). Consistently, *FaMADS1*-over-expressing fruit exhibited lower a* values and total anthocyanin levels relative to the empty vector control (Figure 4D,F). *FaMADS1* over-expression also suppressed fruit softening (Figure 4E). These results indicated that *FaMADS1* inhibited ethylene biosynthesis via down-regulating the expression of *FaSAMS1*, *FaACS1*, and *FaACO1*.

### 2.4. Dual-Luciferase Reporter of FaMADS1 and Ethylene Biosynthesis-Related Genes

**Figure 5:**
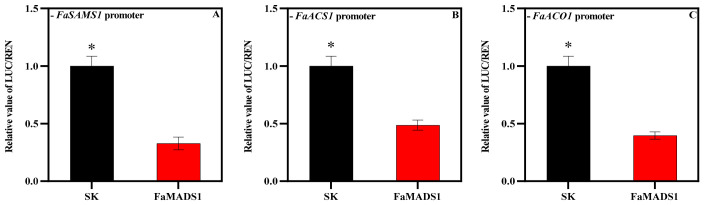
*FaMADS1* suppressed the promoters of *FaSAMS1* (**A**), *FaACS1* (**B**), and *FaACO1* (**C**). SK = empty pGreenII 0029 62-SK effector vector control. Error bars represent standard deviations (SDs) of triplicate biological replicates (n = 3). Prior to Duncan’s multiple range test, Shapiro–Wilk normality test and Levene’s homogeneity of variance test were conducted; all data met normality and homoscedasticity requirements. Significant differences (*p* < 0.05) between treatment groups were determined using Duncan’s multiple range test, denoted by asterisk (*).

To investigate the transcriptional regulatory effects of *FaMADS1* on ethylene biosynthesis genes, we developed a dual-luciferase reporter system assessing *FaSAMS1*, *FaACS1*, and *FaACO1* promoter activities. The *FaMADS1* coding sequence was cloned into pGreenSK (effector), while *FaSAMS1*/*FaACS1*/*FaACO1* promoters drove firefly luciferase expression in reporter constructs. *FaMADS1* significantly repressed all promoters, reducing LUC/REN ratios to 33% (*FaSAMS1*), 49% (*FaACS1*), and 40% (*FaACO1*) of the empty vector controls (Figure 5), establishing its role as a transcriptional suppressor. This experimental evidence demonstrates that *FaMADS1* functions as a transcriptional suppressor regulating key ethylene biosynthesis pathway genes.

## 3. Discussion

The ripening of strawberry fruit entails coordinated biochemical changes in texture and pigmentation [35]. Color development, mediated by anthocyanin biosynthesis, provides a visual proxy for ripening status [39], with total anthocyanin content established as a reliable quantitative metric [40]. Softening constitutes another hallmark event driven by structural modifications in the cell wall matrix [41]. The degradation of cell wall polysaccharides underpins this textural shift, making firmness an essential parameter for evaluating softening [42]. Several previous reports have verified that ethylene is an essential hormone in strawberry fruit ripening [26,33]. Our previous study revealed that ethylene was indispensable for strawberry anthocyanin accumulation [43]. Ethylene is also involved in cell wall degradation to positively regulate ‘Chandler’ strawberry fruit ripening [44]. In this study, the ethylene production of postharvest ‘Akihime’ strawberry fruit increased in 8 d after ABA treatment (Figure 1B), which was consistent with the conclusion of Atta-Aly et al. [45]. Furthermore, our study also clarified that the increase in ethylene production induced the conversion of strawberry fruit from white to red via promoting anthocyanin accumulation (Figure 1A,E). Simultaneously, with ethylene production, the decline of strawberry fruit firmness was accelerated (Figure 1D). Collectively, our data confirmed ethylene as a positive regulator of strawberry fruit ripening, orchestrating key physiological changes such as anthocyanin accumulation and cell wall remodeling.

Beyond color and firmness, fruit ripening regulated by ABA and ethylene is closely coupled with soluble sugar metabolism, organic acid metabolism and flavor formation, which determine the edible quality and market competitiveness of strawberry. Previous research has confirmed that ABA can significantly elevate soluble sugar contents and maintain cell wall structure to extend strawberry postharvest life during storage, while ethylene acts synergistically with ABA to modulate sugar conversion and volatile flavor synthesis [11]. In the present work, we revealed that ABA down-regulated *FaMADS1* to activate ethylene biosynthesis [38]. This ABA-*FaMADS1*-ethylene cascade is speculated to further regulate sugar accumulation, sugar–acid ratio and flavor compound production during fruit ripening and storage [11]. Excess ethylene accumulation induced by ABA may accelerate not only fruit softening but also dynamic changes in taste and flavor during long-term storage, which will ultimately affect overall sensory experience and consumer acceptance. Therefore, fine-tuning the ABA-*FaMADS1* module can potentially balance ripening progress, textural property, flavor quality and shelf life of strawberry fruit.

While the ethylene biosynthesis pathway in fruit is extensively characterized [46,47], it should be noted that the current study mainly focused on the hormonal and molecular mechanism of ABA regulating ethylene biosynthesis via *FaMADS1* and did not systematically determine soluble sugar, organic acid, or the sugar–acid ratio. ABA treatment enhanced ethylene production by up-regulating *FaSAMS1*, *FaACS1*, and *FaACO1* (Figure 2B–D) while suppressing *FaMADS1* (Figure 2A). Complementary evidence from transient assays revealed an inverse relationship: *FaMADS1* silencing elevated ethylene emission (Figure 3A–F), whereas over-expression suppressed it (Figure 4A–F). Critically, dual-luciferase assays confirmed that *FaMADS1* directly represses promoters of *FaSAMS1*, *FaACS1*, and *FaACO1* (Figure 5), identifying *FaMADS1* as a transcriptional inhibitor of ethylene synthesis. It should be noted that the dual-luciferase assay only verifies the overall transcriptional repression of *FaMADS1* against the three ethylene biosynthetic gene promoters and cannot confirm whether *FaMADS1* directly binds to CArG cis-regulatory elements or indirectly inhibits transcription via interacting partner proteins. Direct DNA-binding verification experiments (EMSA and Y1H) and *FaMADS1*-interacting protein screening are currently in progress in our laboratory, and these detailed molecular mechanisms will be systematically reported in our subsequent independent research article.

**Figure 6:**
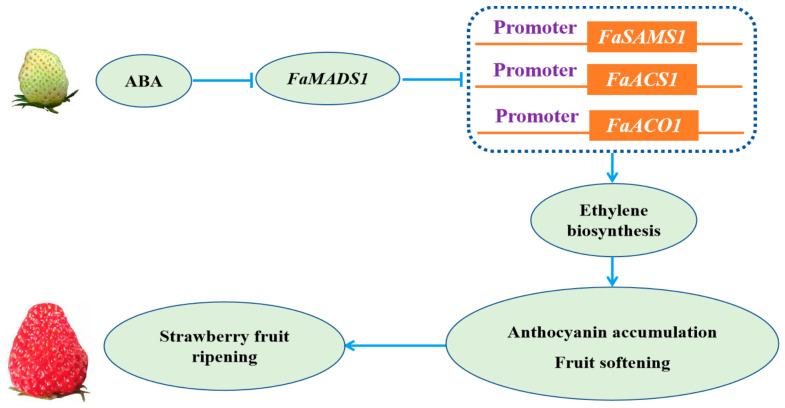
ABA–ethylene crosstalk in strawberry ripening. ABA signal suppressed the expression of transcription factor *FaMADS1*, while *FaMADS1* inhibited the expression of *FaSAMS1*, *FaACS1*, and *FaACO1* expression through repressing their promoters. Then, ethylene was synthesized. Ethylene accelerated strawberry fruit ripening via promoting anthocyanin accumulation and fruit softening.

The hormonal coordination of ripening requires precise phytohormone interplay. Our findings reveal that ABA synchronizes with ethylene via *FaMADS1*: ABA down-regulates this transcription factor (Figure 2A), de-repressing ethylene biosynthetic genes (*FaSAMS1*, *FaACS1*, *FaACO1*) and elevating ethylene output (Figure 1B). This extends beyond established ABA signaling networks and hormonal synergies [48]. As depicted in Figure 6, ABA-induced *FaMADS1* suppression propagates ethylene synthesis, which in turn mediates anthocyanin accrual and textural softening to drive ripening completion. It should be noted that the current study mainly focused on the hormonal and molecular mechanism of ABA regulating ethylene biosynthesis via *FaMADS1* and did not systematically determine soluble sugar, organic acid, sugar–acid ratio and flavor volatile components. Combined with published studies, we propose that subsequent research should further explore how the ABA-*FaMADS1*-ethylene pathway regulates sugar and flavor metabolism and evaluate the comprehensive quality and consumer acceptability of ABA-treated strawberry fruit under simulated commercial storage conditions. Such works will further complement the regulatory network of strawberry ripening and provide more comprehensive theoretical support for the application of ABA in postharvest preservation.

## 4. Materials and Methods

### 4.1. Fruit Material and Treatments

The experimental material comprised octoploid strawberry (*Fragaria* × *ananassa* Duch. cv. Akihime) obtained from a plantation in Wenzhou, Zhejiang, China. Following established protocols for developmental stage selection, white-stage fruits (W stage, 19 DAA) exhibiting uniform morphometric parameters were collected and immediately transferred under controlled conditions to the laboratory facility [19].

Prior to experimental treatments, all fruits underwent standardized surface sterilization involving sequential immersion in 0.5% (*v*/*v*) sodium hypochlorite solution followed by rinsing with sterile distilled water. Using stratified randomization, 180 fruits were allocated into three experimental cohorts: (1) ABA treatment (1 mM), (2) NDGA treatment (1 mM), and (3) aqueous control. Each group underwent immersion in respective solutions for 120 s at 25 ± 1 °C, with subsequent surface moisture removal via sterile absorbent paper. Processed samples were maintained under controlled postharvest conditions (20 °C, RH 85%) for subsequent biochemical analyses.

### 4.2. Ethylene Emission

Ethylene quantification was performed using static-chamber gas chromatography, following established protocols with modifications. Ten intact fruit specimens were equilibrated in 2 L hermetically sealed containers at 20 ± 0.5 °C for 2 h to allow gas phase stabilization. Headspace sampling was conducted using gas-tight syringes (Hamilton, NV, USA), with 2 mL aliquots injected into a GC-2030 gas chromatograph (Shimadzu, Kyoto, Japan). Quantitative analysis was achieved through external calibration using certified ethylene standards (Sigma-Aldrich, Shanghai, China; 10 ppm ± 5% accuracy). Ethylene evolution rates were normalized to fresh weight and expressed as ng kg^−1^ h^−1^ [49].

### 4.3. Fruit Color, Firmness, and Total Anthocyanin Content (TAC)

Fruit quality parameters including chromaticity, textural properties, and total anthocyanin content (TAC) were quantified using established protocols. Colorimetric measurements (Konica Minolta CR-200, Tokyo, Japan) were performed at two equatorial positions per fruit, with the a* value adopted as the chromaticity indicator. Parallel textural analysis utilizing a TA-XT2i texture analyzer was conducted on identical sampling sites to determine firmness. For phytochemical characterization, TAC was determined following Chen’s spectrophotometric methodology [43], whereas color and firmness assessments referenced Li’s operational framework [31].

### 4.4. RNA Extraction, cDNA Synthesis, and RT-qPCR

Total RNA was isolated from strawberry fruit using a modified CTAB method [43], incorporating β-mercaptoethanol during homogenization and chloroform–isoamyl alcohol for phase separation. Nucleic acid purity was assessed spectrophotometrically, with OD260/OD280 ratios (1.8–2.0) confirmed using a 10 M multimode microplate reader (Tecan, Zurich, Switzerland; SPARK^®^). First-strand cDNA was synthesized from 1 μg of total RNA employing HiScript III All-in-one RT SuperMix (Vazyme, Nanjing, China; R323-01) under thermal conditions of 42 °C for 15 min and 85 °C for 5 s. Transcript abundance was quantified via SYBR Green-based qRT-PCR (Roche, Nanjing, China; LightCycler^®^ 480 II) using three biological replicates. Amplification specificity was validated by melt curve analysis and electrophoretic confirmation of amplicon size. The *FaActin* gene (GenBank: AB116565) served as the endogenous normalization control across all samples.

### 4.5. Plasmid Construction and Agro-Infiltration

*Tobacco rattle virus* (pTRV1 and pTRV2) VIGS vectors were selected to silence *FaMADS1,* and pGreenII 0029 62-SK vector (SK) was considered an over-expression vector in this study. The coding sequence of *FaMADS1* was inserted into the BamHI and EcoRI restriction sites of pTRV2 vector and the SmaI and EcoRI restriction sites of SK vector, respectively. All vectors were then transferred into *Agrobacterium tumefaciens* (strain EHA105). *A. tumefaciens* were injected into strawberry fruit at the W stage through the stalk [50].

### 4.6. Dual-Luciferase Reporter

To elucidate the regulatory effects of *FaMADS1* on ethylene biosynthesis components (*FaSAMS1*, *FaACS1*, *FaACO1*), we engineered two modular constructs: (i) effector vectors containing the full-length *FaMADS1* cDNA cloned into pCAMBIA1300-SK (KanR) and (ii) reporter vectors with individual promoters (*FaSAMS1*p::LUC, *FaACS1*p::LUC, *FaACO1*p::LUC) inserted into pGreenII 0800 (AmpR) [51]. Through electroporation-mediated transformation, these constructs were introduced into *Agrobacterium tumefaciens* EHA105 competent cells. Transconjugants were selected on LB medium containing 50 μg/mL rifampicin and 100 μg/mL kanamycin. For transient expression assays, bacterial suspensions (OD600 = 0.6) carrying effector/reporter pairs were co-infiltrated into the abaxial surface of 6-week-old *Nicotiana benthamiana* leaves using needless syringes. After 72 h post-infiltration in controlled growth chambers (22 °C, 16/8 h photoperiod), leaf discs (10 mm diameter) were excised for luminescence quantification. Using the Dual-Luciferase^®^ Reporter Assay System (Vazyme, Nanjing, China; Promega, E1910), firefly luciferase (experimental signal) and Renilla luciferase (internal control) activities were sequentially measured with a GloMax^®^ Navigator Microplate Luminometer (Tecan, Zurich, Switzerland). Normalized regulatory activity was calculated as FLUC/RLUC ratio ± SD from three biological replicates.

The Renilla luciferase (REN) reporter included in every pGreenII 0800 reporter vector (Miaoling Biotechnology Co., Ltd., Wuhan, China) acts as an intrinsic internal reference to offset variations in agroinfiltration efficiency, leaf physiological status and sampling deviation among different leaf discs. Uniform infiltration conditions (consistent OD600, leaf position, culture environment and incubation time) were applied to all samples to further minimize experimental bias. Empty pGreenII 0029 62-SK vector (Miaoling Biotechnology Co., Ltd., Wuhan, China) co-infiltrated with each promoter-LUC reporter was used as the baseline control to reflect intrinsic basal promoter activity, a standard control design in tobacco dual-luciferase transient expression systems. Renilla luciferase (REN) served as an internal standard to normalize differences in agroinfiltration efficiency across leaf samples, and all regulatory activity was calculated as normalized FLUC/RLUC ratios to eliminate variability from uneven transformation efficiency.

### 4.7. Statistical Analysis

All data were presented as the mean ± standard deviation (SD) with three biological replicates (n = 3). Before ANOVA and Duncan’s multiple range test, Shapiro–Wilk test was used to verify data normality, and Levene’s test was applied to check homogeneity of variances. All datasets satisfied normal distribution and homogeneous variance assumptions, so factorial ANOVA followed by Duncan’s multiple range test (*p* < 0.05) was performed using SPSS Statistics 26.0 (IBM Corp., San Jose, CA, USA). Significant differences among treatments were marked with distinct lowercase letters (a–c).

## 5. Conclusions

This investigation provides evidence that ethylene biosynthesis in postharvest strawberry fruit was promoted by ABA through transcriptional regulation of *FaMADS1*. During fruit ripening, enhanced ethylene production showed a positive correlation with exogenous ABA application. Molecular analyses revealed an inverse relationship between *FaMADS1* expression and ethylene metabolic activity, where ABA down-regulated *FaMADS1* transcription, concurrently alleviating its suppressive effects on (a) ethylene biosynthesis pathways and (b) transcript abundance of key genes (*FaSAMS1*, *FaACS1*, and *FaACO1*). These findings elucidate a previously uncharacterized regulatory network governing the hormonal crosstalk between ABA and ethylene during postharvest physiological processes in strawberry. Notably, this work only characterizes the ABA-*FaMADS1*-ethylene regulatory cascade without profiling soluble sugars, organic acids, the sugar–acid balance or flavor volatiles. Future research will dissect how this module governs flavor metabolism and fruit sensory quality under simulated cold-chain storage to enrich strawberry ripening regulatory networks and advance ABA-based postharvest preservation strategies.

## Data Availability

The original contributions presented in this study are included in the article/Supplementary Material. Further inquiries can be directed to the corresponding author.

## Supplementary Materials

The following supporting information can be downloaded at: https://www.mdpi.com/article/10.3390/plants15142202/s1, Table S1. Primers used in this study.
