# Supplementary material for: Abscisic Acid Stimulates Ethylene Biosynthesis by Repressing the Expression of FaMADS1 in Postharvest Strawberry Fruit
Source: Plants (Basel). 2026 Jul 19;15(14):2202. doi: 10.3390/plants15142202 (PMC13417630; doi:10.3390/plants15142202)
Supplement: Supplementary file 1 [file plants-15-02202-s001.zip › plants-4381149-supplementary.pdf]

plants

Supplementary Materials

Table S1 Primers used in this study

Gene name	Forward primers (5′-3′)	Reverse primers (5′-3′)
FaActin	TGGGTTTGCTGGAGATGAT	CAGTAGGAGAACTGGGTGC
FaMADS1-RT	AACTTTAGACAAACTTGGGAAC	GGCTGGTGTGGCTGTAGGCAT
FaSAMS1-RT	ACATTGAGCAGCAGAGC	GGGTTTCATCAGTGGC
FaACS1-RT	CTTACCATCCCACCACCAAC	TTCTTTTGCTCCTTGTGCTG
FaACO1-RT	TACCTCAAGCACCTTCCTCGC	TTAGTGCCAAAGGTAGGACTA
FaMADS1-RNAi	TGCGGATCCATGGGGAGGGGAA-GAGTGG	CCGGAATTCTCAGAGCATCCAAC-CAGG
FaMADS1-OE	TGCAAGCTTATGGGGAGGGGAA-GAGTGG	CCGGGATCCTCAGAGCATCCAAC-CAGG
FaSAMS1-*pro*-LUC	AAGCTTATGTATTGTTCATTTTT-GTTTTCCAGT	GGATCCTTCTGCTACAAAGAA-TAGAACAAAAAAA
FaACS1-pro-LUC	AAGCTTTT-GAGCTGGTGAGCCATGGG	GGATCCCTGACCTTGTCATCTT-GGAACAGC
FaACO1-pro-LUC	AAGCTTTTGAGTTGGTGAGTCATGG-GATAC	GGATCCAGGCTTCATGTAG-TCATCGAACAC
FaMADS1-SK	CCCGGGATGGGGAGGGGAAGAG-TGG	GAATTCTCAGAGCATCCAACCAGG
